# Antibodies Against SARS-CoV-2 Do Not Cross-React with Endemic Coronaviruses in a Pediatric Population: Data from a Bangladesh Cohort

**DOI:** 10.3390/v17020161

**Published:** 2025-01-24

**Authors:** Ana Citlali Márquez, Guadalein Tanunliong, Mamun Kabir, Masud Alam, Biplob Hossain, Humaira Rashid, Agatha N. Jassem, Inna Sekirov, Rashidul Haque, Muhammad Morshed

**Affiliations:** 1 BCCDC Public Health Laboratory, Vancouver, BC V5Z 4R4, Canada; 2 Department of Pathology and Laboratory Medicine, University of British Columbia, Vancouver, BC V6T 1Z4, Canada; 3 International Centre for Diarrhoeal Disease Research, Dhaka 1212, Bangladesh

**Keywords:** SARS-CoV-2, COVID-19, HCoVs

## Abstract

There is a limited understanding of the immunological differences between children and adults that protect children from developing severe coronavirus disease 2019 (COVID-19) following SARS-CoV-2 infection. Previous infection with endemic human coronaviruses (HCoVs) has been suggested as a factor. In this study, we used 100 paired residual samples collected before and during the COVID-19 pandemic from children in Bangladesh. We compared the changes in their sero-status (no COVID-19 vs. COVID-19) and quantified antibody levels to HCoVs. We found that although 45% of the children seroconverted for IgG antibodies against SARS-CoV-2, there was no correlation between evidence of previous infection with HCoVs and the magnitude of SARS-CoV-2 antibody responses post-infection. Moreover, no differences in the anti-HCoV antibody levels were found pre- and post-SARS-CoV-2 infection.

## 1. Introduction

Before the surge of coronavirus disease 2019 (COVID-19), caused by severe acute respiratory syndrome coronavirus 2 (SARS-CoV-2), there were six known coronaviruses with the ability to infect humans [1]. HCoV-NL63, HCoV-229E (alphacoronaviruses), and HCoV-OC43 and HKU1 (beta-coronaviruses) are considered common and widely circulating childhood infections that cause mild infections in the upper respiratory tract [2]. MERS-CoV and SARS-CoV-1 do not circulate widely, although they are highly pathogenic [1].

SARS-CoV-2 infection in children is often mild or asymptomatic [3], though the mechanisms underlying this remain unclear [4]. While antibodies produced after endemic human coronavirus (HCoV) infections can cross-react with SARS-CoV-2 in children [5,6], such protection primarily confers homotypic immunity, with limited evidence of heterotypic immunity [7,8]. However, it has been suggested that differences in HCoV exposure and variations in immune responses in between children and adults may play an important role in the clinical outcomes after SARS-CoV-2 infection [4,9,10].

This study aimed to examine antibody responses to SARS-CoV-2 and HCoVs both prior to the COVID-19 surge and during the initial wave of the pandemic among children in Bangladesh. For this, we used residual samples collected in Bangladesh before and after the surge of COVID-19 to determine the prevalence of SARS-CoV-2 infection in children between 4 and 6 years of age from the region of Mipur, Dhaka.

## 2. Materials and Methods

### 2.1. Samples

Plasma specimens were collected as part of the study “Field Studies of Cryptosporidiosis and Enteropathogens in Bangladesh” (PR-13092). The cohort consisted of plasma specimens longitudinally collected between March and October 2019 (pre-COVID-19 pandemic) from children aged 4–5 years old (*n* = 100) and between September and October 2020 (during COVID-19) from the same children (5–6 years old, *n* = 100). Samples were shipped to the British Columbia Centre for Disease Control, Public Health Laboratory (BCCDC-PHL) from the International Centre for Diarrhoeal Disease Research, Bangladesh (icddr,b) following IATA regulations.

### 2.2. Antibody Testing

Antibody detection was performed using the V-PLEX COVID-19 Coronavirus Panel 2 (IgG) from Meso Scale Discovery (MSD). This assay detects IgG antibodies against nine antigens: the Spike, Nucleocapsid, and (Receptor Binding Domain) RBD proteins of SARS-CoV-2 (Wuhan strain), and antibodies against the Spike proteins of SARS-CoV-1, HCoV-229E, HCoV-HKUI, HCoV-OC43 and HCoV-NL63.

Plasma samples were diluted to 1:5000 concentration, and the assay was performed according to manufacturer’s instructions (#K15368U). Plates were read using the MSD QuickPlex SQ120, and initial analysis was performed on MSD’s Discovery Workbench 4.0 software. Interpretation of SARS-CoV-2 antibody status was performed on R (Version 4.1.2), using the following SARS-CoV-2 reactivity cut-offs: SARS-CoV-2 Spike > 1960 AU/mL, Nucleocapsid > 5000 AU/mL and S1 RBD > 538 AU/mL. Samples with reactivity to at least two out of three antigens were considered positive against SARS-CoV-2 [1]. Seroconversion plots were created on GraphPad Prism 9.

### 2.3. Statistical Analysis

Bonferroni-adjusted Wilcoxon signed-rank and Wilcoxon rank-sum tests, multivariable regression modeling, and visualization of processed data were all carried out on R (Version 4.1.2) using the stats (Version 4.1.2), ggplot2 (Version 3.3.5) packages. Model diagnostics were assessed to confirm that the assumptions for regression models were met prior to building multivariable linear regression models. Non-significant predictor variables were kept in the model for face validity. *p*-values less than 0.05 were considered statistically significant.

## 3. Results

### 3.1. Seroconversion to SARS-CoV-2 in This Pediatric Bangladeshi Cohort Following the COVID-19 Surge Was 45%

**Figure 1:**
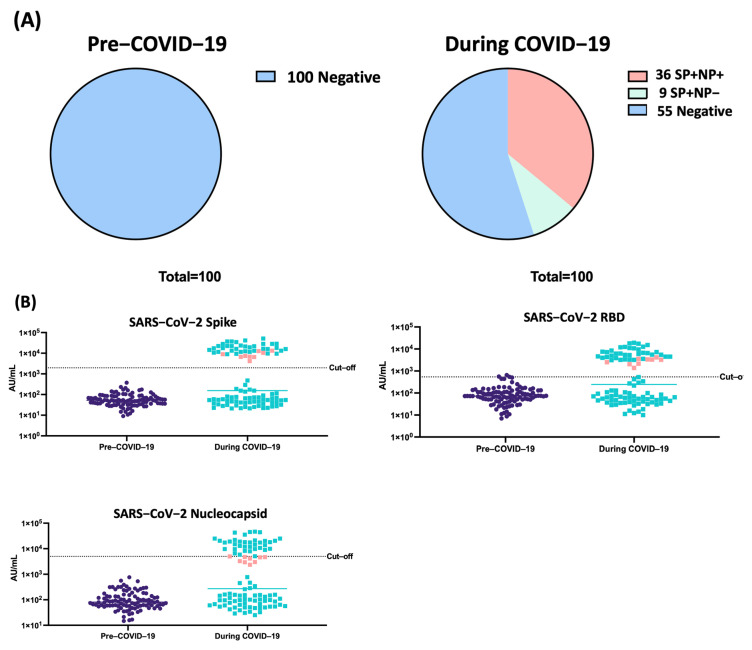
A total of 45% of Bangladeshi children tested during COVID-19 have antibodies against SARS-CoV-2. Samples were tested for antibodies against SARS-CoV-2 Nucleocapsid, Spike and RBD. Reactivity to two out of three antigens was considered positive for SARS-CoV-2 infection. (**A**) Summary of positive and negative samples on the Bangladesh cohort. (**B**) Values were plotted depending on their time of collection: pre-COVID-19 and during COVID-19. Dotted lines mark reactivity cut-offs determined for the assay. Pink squares denote samples for which Nucleocapsid levels were below the cut-off, while anti-Spike and anti-RBD levels were above the cut-off.

We determined the prevalence of infection in children from the Bangladeshi cohort using the MSD multiplex assay. We first tested the samples collected before the surge of COVID-19 (Figure 1A). All 100 samples were negative on the interpretation algorithm. Next, we tested samples from the same children collected in 2020, during the surge of the COVID-19 pandemic.

We found that of the 100 samples, 45% (45/100) came back positive for anti-SARS-CoV-2 antibodies. Of these, 39 samples were above the cut-offs for the three antigens, while 9 samples were positive for Spike and RBD only (Figure 1B).

Given that, at the time of collection, no vaccine had been developed, the antigens present in the samples could only have come from exposure to the virus. These results suggest that 45% of the children had been in contact with SARS-CoV-2 by the end of 2020.

### 3.2. No Differences in HCoV Levels Between SARS-CoV-2 Seronegative and Seropositive Individuals

We explored whether previous infection with HCoVs had any effect on antibody levels against SARS-CoV-2 amongst those seropositive for COVID-19 (N = 45). Regression analysis demonstrates that pre-pandemic anti-HCoV antibody levels are not significantly associated with corresponding SARS-CoV-2 IgG antibody levels post-seroconversion (Table 1).

**Figure 2:**
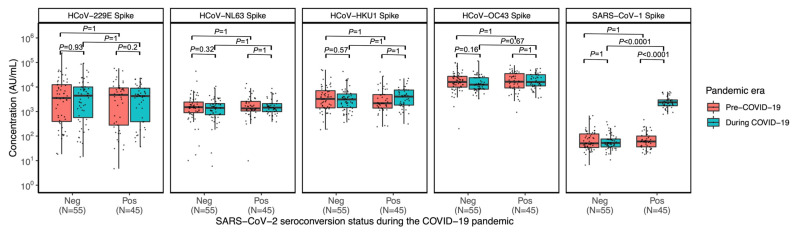
Longitudinal IgG antibody levels against Spike of HCoV-229E, HCoV-NL63, HCoV-HKU1, and HCoV-OC43 and SARS-CoV-1 among children aged 4–6 before and during the COVID-19 pandemic. Participants were stratified based on their sera collected during the COVID-19 pandemic and stratified as SARS-CoV-2 seropositive (N = 45) or seronegative (N = 55). Bonferroni-adjusted Wilcoxon rank-sum test was used to compare sera collected either before or during the COVID-19 pandemic between seronegative vs. seropositive individuals. Wilcoxon signed-rank test with Bonferroni correction was used to compare serum IgG levels in paired longitudinal sera of both seronegative and seropositive individuals. Box and whisker plots denote median and interquartile ranges. Significance level set at alpha = 0.05.

Next, we examined if infection with SARS-CoV-2 would boost the antibody levels of other coronaviruses. Individuals were grouped according to their immune response status to SARS-CoV-2 in the sera collected during the COVID-19 pandemic: either seropositive (N = 45) or seronegative (N = 55). When comparing their paired pre- and during-pandemic longitudinal sera, no significant changes in IgG antibody levels against HCoV-229E, HCoV-NL63, HCoV-HKU1, and HCoV-OC43 were observed following seroconversion to SARS-CoV-2, compared to their own pre-pandemic baseline HCoV antibody levels (Figure 2). When comparing the anti-HCoV antibody levels at the two collection timepoints, between individuals that remained seronegative (N = 55) and individuals that became seropositive (N = 45) to SARS-CoV-2, no significant difference in HCoV IgG antibody levels between the two groups was detected, both before and during the COVID-19 pandemic (Figure 2). However, we observed elevated SARS-CoV-1 Spike antibodies amongst those seropositive for SARS-CoV-2, compared to those seronegative during the pandemic (*p* < 0.0001) and compared to their baseline serum levels collected pre-pandemic (*p* < 0.0001) (Figure 2), which suggests cross-reactivity between SARS-CoV-1 and SARS-CoV-2 Spike antibodies.

## 4. Discussion

Previous recent infections with HCoVs were originally suspected as one of the reasons for children presenting a mild/asymptomatic disease when infected with SARS-CoV-2.

Plasma specimens collected longitudinally before and during the COVID-19 pandemic allowed us to gain insight into changes in the antibody response to endemic coronaviruses and SARS-CoV-2 before and after SARS-CoV-2 infection.

We measured anti-SARS-CoV-2 antibody levels (IgG) in children between the ages of 4–6 years in a cohort collected in Dhaka, Bangladesh. Our results show that following the COVID-19 surge, as many as 45% of the sampled individuals showed evidence of SARS-CoV-2 exposure. Specifically, 36% (36/100) of the samples tested positive for antibodies against SARS-CoV-2 Spike, RBD and Nucleocapsid, while an additional 9% (9/100) of samples were positive for antibodies against SARS-CoV-2 Spike and RBD only. While some children with antibodies against SARS-CoV-2 S1 Spike and RBD did not have antibodies against SARS-CoV-2 Nucleocapsid above the cut-off, these samples had higher levels of Nucleocapsid than uninfected individuals, and their levels were just below the positivity cut-off threshold. Since these samples were collected in 2020, before the commercialization of any COVID-19 vaccine, the low level of anti-Nucleocapsid antibody could be reflective of antibody waning. For the purposes of this analysis, we considered samples positive for 2 out of 3 anti-SARS-CoV-2 antibodies as showing evidence of SARS-CoV-2 infection.

Like others [8,9,11,12], we did not observe any correlation between baseline HCoV levels and antibody levels in response to SARS-CoV-2 among seropositive individuals (Table 1). This suggests that, at least at the antibody level, previous infection with HCoVs does not impact the immune response to SARS-CoV-2 in children.

Our prior work, as well as that of others [7,13,14], has indicated a potential cross-reactivity between antibodies against SARS-CoV-2 Spike and SARS-CoV-1 Spike, as well as increased levels in antibodies to beta-HCoVs in individuals with a history of SARS-CoV-2 infection. In this cohort, we see a positive correlation between SARS-CoV-2 infection and SARS-CoV-1 cross-reactive antibodies; however, this correlation is not present with any of the other HCoVs. This suggests differences in the immune response to coronaviruses in young children relative to older individuals. These findings could be an indication that this cohort of children had not encountered beta-coronaviruses prior to collection of both samples, or had a significant waning of anti-beta-coronavirus immunity by the time of collection. Nevertheless, these scenarios seem unlikely given the global ubiquity of HCoVs and how common infections in children are [15,16,17,18]. Furthermore, the observed levels of anti-HCoVs antibodies are unlikely to be a result of non-specific cross-reactive binding.

A limitation of his study is the relatively small size of the cohort and the inability to determine the relative timeline between HCoVs and SARS-CoV-2 infections which restrict the generalizability of the findings. To our knowledge, no studies have yet been conducted to assess antibody cross-reactivity between HCoVs and SARS-CoV-2 variants in children. Despite these limitations, we were able to determine that by the Fall of 2020, a high proportion of young children in our cohort had already been exposed to SARS-CoV-2. Our results indicate that previous infection with HCoVs does not influence the magnitude of antibody response to SARS-CoV-2 and provide supporting evidence that humoral response against HCoVs might not account for a mild pediatric presentation of SARS-CoV-2 infection. Finally, we see evidence that after SARS-CoV-2 infection in young children, there is no antibody boosting against HCoVs, suggesting that there is no additional layer of protection against future infections with HCoVs in young children. However, further studies are necessary to confirm if similar outcomes are observed given the impact of emerging variants and advancements in vaccine development. Future research should prioritize recruiting a larger cohort of children with documented SARS-CoV-2 infections and vaccination histories, including younger children who have not yet been exposed to HCoVs, to establish a more robust baseline for comparison.

## Figures and Tables

**Table 1:** Multiple linear regression analysis of anti-SARS-CoV-2 Spike, RBD, and Nucleocapsid IgG antibody levels in SARS-CoV-2 seropositive individuals (N = 45) following seropositivity, with adjustment for age, and pre-pandemic (baseline) anti-HCoV IgG concentration.

Variable	SARS-CoV-2 Spike		SARS-CoV-2 RBD		SARS-CoV-2 Nucleocapsid	
	Est	*p*	Est	*p*	Est	*p*
(Intercept)	8.9	-	7.55	-	10	-
Age	–0.05	0.74	–0.06	0.76	–0.27	0.31
log(HCoV-229E Spike)	0.05	0.19	0.07	0.08	0.02	0.74
log(HCoV-HKU1 Spike)	0.07	0.41	0.12	0.23	0.05	0.71
log(HCoV-NL63 Spike)	–0.09	0.25	0.06	0.49	0.09	0.44
log(HCoV-OC43 Spike)	–0.07	0.06	–0.05	0.64	–0.04	0.82
Adjusted R-squared	–0.024		0.013		–0.07	

## Data Availability

The raw data supporting the conclusions of this article will be made available by the authors on request.
